# Early clearance versus control: what is the meaning of a negative tuberculin skin test or interferon-gamma release assay following exposure to *Mycobacterium tuberculosis*?

**DOI:** 10.12688/f1000research.13224.1

**Published:** 2018-05-25

**Authors:** Erin W. Meermeier, David M. Lewinsohn

**Affiliations:** 1 Pulmonary and Critical Care Medicine, Department of Medicine, Oregon Health and Science University, Portland, USA; 2 Department of Medicine, VA Portland Health Care System, Portland, OR, USA

**Keywords:** Mycobacterium tuberculosis, preventative vaccine, infection, household contact studies

## Abstract

The elimination of tuberculosis (TB) cannot reasonably be achieved by treatment of individual cases and will require an improved vaccine or immunotherapy. A challenge in developing an improved TB vaccine has been the lack of understanding what is needed to generate sterilizing immunity against *Mycobacterium tuberculosis* (Mtb) infection. Several epidemiological observations support the hypothesis that humans can eradicate Mtb following exposure. This has been termed early clearance and is defined as elimination of Mtb infection prior to the development of an adaptive immune response, as measured by a tuberculin skin test or interferon-gamma release assay. Here, we examine research into the likelihood of and possible mechanisms responsible for early clearance in household contacts of patients with active TB. We explore both innate and adaptive immune responses in the lung. Enhanced understanding of these mechanisms could be harnessed for the development of a preventative vaccine or immunotherapy.

## Introduction

Tuberculosis (TB) is a leading cause of infectious disease mortality worldwide, accounting for approximately 6.3 million new cases and 1.4 million deaths in 2016 (World Health Organization [WHO] TB Report 2017). The recent emergence of strains of *Mycobacterium tuberculosis* (Mtb) resistant to nearly all effective drug therapy highlights the need for alternative strategies to TB control. The Bacille Calmette–Guérin (BCG) vaccine has been widely used but is controversial, as its efficacy for the prevention of adult TB has been variable. Moreover, despite the widespread use of the BCG vaccine worldwide, the incidence of TB has not dramatically decreased. The elimination of TB cannot reasonably be achieved by treatment of individual cases and will require an improved vaccine or early detection of those likely to progress ^1, 2^. The main challenge in developing an improved TB vaccine has been the lack of understanding correlates of protective immunity. What is needed to generate sterilizing immunity against Mtb infection, unlike in many other preventable infectious diseases, is still unknown.

Mtb is transmitted via the aerosol delivery of small particulates into the lung. Here, a dynamic interplay between host immune mechanisms and the virulent bacterium begins with the innate immune system. Innate immunity provides functional and immediate defenses against microbial infection through shared germline-encoded processes and receptors. In addition to the direct recognition and control of microbial infection, innate immunity plays a central role in the initiation and maintenance of subsequent adaptive immune responses. In the lung, Mtb can interact with and infect different cell types, including macrophages, and can establish stable intracellular infection ^3^. When infection overcomes initial physical and immunological defenses, exposure to pathogen-derived antigens leads to an adaptive immune response over the ensuing two to six weeks. This response relies upon clonal expansion of antigen-specific lymphocytes and forms the basis for immunological memory ^3^. Tuberculin skin tests (TSTs) and interferon-gamma release assays (IGRAs) measure immune sensitization to Mtb and can reflect whether infection has occurred. Individuals who test positively with a TST or IGRA but have no evidence of TB are considered to have latent tuberculosis infection (LTBI). Among LTBI individuals, the estimated lifetime risk of developing TB is roughly 10% (WHO Global TB Report 2016). Neither the TST nor the IGRA can serve as a reflection of bacterial burden ^4^. Nonetheless, in the setting of a household exposure, both tests have excellent negative predictive values in that they predict those who are unlikely to get TB ^5, 6^.

**Figure 1.**
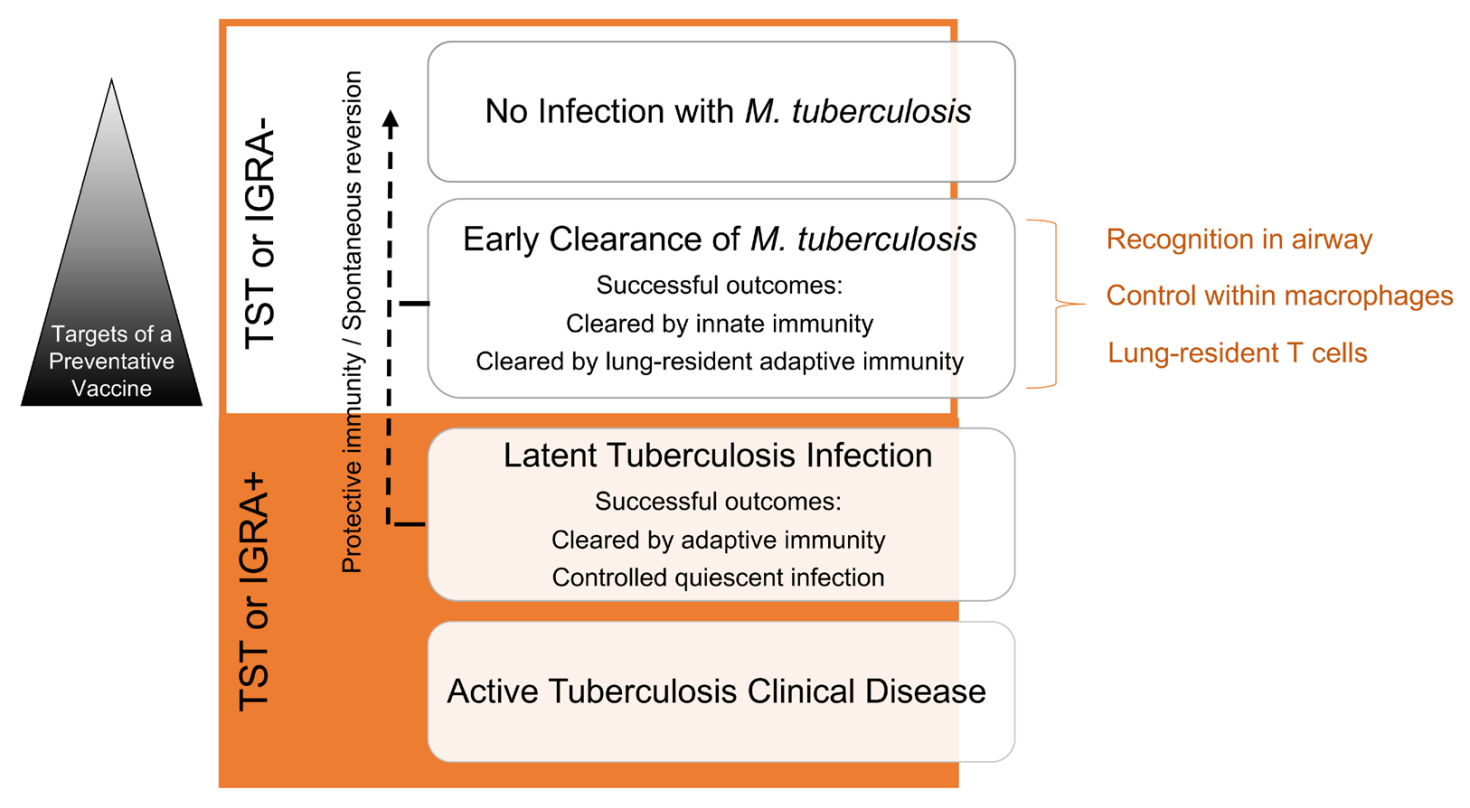
Outcomes of exposure to *Mycobacterium tuberculosis* (Mtb). Mtb exposure can lead to infection, early clearance, latent tuberculosis infection, or, ultimately, tuberculosis. Early clearance occurs before the development of an adaptive immune response detected by a tuberculin skin test (TST) or interferon-gamma release assay (IGRA). Understanding of the underlying mechanisms dictating the outcomes of exposure to Mtb could greatly facilitate the definition of correlates of protective immunity to Mtb infection and generate targets of a preventative vaccine. Possible mechanisms responsible for early clearance in household contacts of active tuberculosis patients that are explored in this review are listed on the right (orange).

Upon exposure to Mtb, a number of disparate outcomes can occur. These outcomes include no infection, early clearance of infection, LTBI, and TB ( Figure 1). Although about one-quarter of the global population is thought to be infected with Mtb, these epidemiological observations indicate that humans have evolved mechanisms to control or eradicate infection with Mtb. First, some individuals repeatedly exposed to Mtb never demonstrate evidence of immune sensitization by TST or IGRA. These individuals may have been able to eradicate Mtb ** with an effective innate immune response before an adaptive immune response develops. This phenomenon is termed early clearance. Second, a subset of individuals with a positive TST or IGRA have been observed to revert to a negative result over time ^7^. Reversion of these tests may indicate a decrease in bacterial load or clearance of infection and is suggestive of control of Mtb infection. Third, of the individuals considered to have LTBI, most contain infection without developing TB, indicating the development of protective immunity. Although those with LTBI are at risk of reactivation, studies of nursing and medical students in the early twentieth century suggest that a history of infection with Mtb correlates with less risk of TB over a lifetime in a subset of individuals. This suggests that prior exposure to Mtb could be protective ^8, 9^. Evidence to support these three cases of resistance to Mtb has been highlighted in two reviews published in 2014 ^10, 11^. Nevertheless, early clearance of Mtb infection has been the focus of intensified research over the past three years. Understanding of these events could greatly facilitate the definition of correlates of protective immunity to Mtb infection.

In this commentary review, we present and summarize recent research advances in understanding the epidemiology of early clearance of Mtb infection. We include studies of loci of resistance, mechanisms of early clearance, and natural transmission model systems and instigate discussion of the relevance of these advances to vaccines to control infection by Mtb.

## Evidence for early clearance

A difficulty in studying early clearance of Mtb infection has been discerning it from non-exposure, as lack of converting a TST or IGRA could reflect either situation. In the absence of a gold standard for diagnosing Mtb infection, it is possible that either test could miss instances of infection. However, given the excellent negative predictive value of both tests, we will consider either negative test as a reflection of resistance to infection ^5, 6^. Nevertheless, longitudinal studies of household contacts (HCs) of active TB cases have allowed for the definition of risk factors of Mtb infection. For example, a Uganda–Case Western Reserve University research collaboration has been conducting a long-standing study of HCs since 1995. Recently compiled data from this study were used to define risk factors of Mtb infection as measured by TST positivity in over 1,300 individuals. Among adult HCs, there were no significant differences in a multifactorial risk score between individuals who were persistently TST-negative and those who had a positive TST or converted to a positive TST over two years ^12^. This result was further supported by a culminating analysis of over 2,500 HCs from the same cohort ^13^. Here, persistently TST-negative adults did not differ in epidemiologic risk score from other clinical groups. These data suggest that there is still evidence for early clearance even when the degree of exposure is controlled for.

A number of different studies have tried to establish the prevalence of early clearance. Here, the prevalence of early clearance of infection can be defined by a persistent negative TST or IGRA in the setting of Mtb exposure. In humans, our best examples of early clearance come from longitudinal (two-plus years) analyses of HCs of TB patients, or those residing in high TB-burden areas, where immune sensitization is carefully monitored. Table 1 and Table 2 summarize longitudinal studies of HCs categorized as persistently TST-negative ( Table 1) or persistently TST- or IGRA-negative or both ( Table 2). As illustrated in Table 1, studies with at least two years of longitudinal observation of HC conversion of TST demonstrated a frequency of early clearance ranging from 3.4% ^14^ to 26.8%, both in Uganda ^15^. Whereas the TB Network study that followed HC conversion of IGRA demonstrated that about 58% of exposed HCs lacked evidence of immune sensitization, other studies had a less rigorous definition, as they varied by time of follow-up with individual as well as the likelihood of exposure to TB ^16– 20^. Presumably, in some of these studies, higher rates of TST- and IGRA-negative HCs could be explained by diminished exposure to Mtb. Nonetheless, extensive epidemiological information collected from the studies in Table 1 and Table 2 suggests that when exposure variables are controlled for, there remains a population of individuals who may be better able to control infection ^12, 15, 21, 24– 27^. In this review, we explore possible determinants of early clearance of Mtb infection.

**Table 1.** Studies that have determined persistent tuberculin skin test negativity in household contacts.

Location	Duration of observation	Household contacts	Percentage TST-negative	Reference
Uganda ^a^	2 years	97	26.8	15
Uganda ^a^	2 years	2,585	9.9	21
Tanzania and Uganda	Up to 8 years	469 ^c^	48	22
Venezuela ^b^	3 years	102 ^d^	18.6	23
Uganda ^a^	2 years	601	14.5	24
The Gambia ^b^	6 months	64	60	20
Uganda ^a^	2 years	1,318	11.7	12
Uganda ^a^	1–2 years	529	3.4	25
South Africa	None	350	40	14
Pakistan	2 years	93	25	26
Ghana	None	2,346	5.5	19
Uganda ^a^	2 years	803	10.5	27

^a^Subjects were from the same cohort; ^b^not a high tuberculosis burden area; ^c^all patients were HIV-positive and only 46% of persons enrolled were household contacts; ^d^tuberculosis hospital workers, not household contacts. TST, tuberculin skin test.

**Table 2.** Studies that have determined persistent interferon-gamma release assay negativity in household contacts.

Location	Duration	Test for *Mycobacterium* *tuberculosis* exposure	Household contacts	Percentage IGRA-negative	Reference
Brazil	8–12 weeks	TST and IGRA	838	30.2	16
USA ^a^	8–10 weeks	TST and IGRA	569	52	17
Brazil	1 year	TST and IGRA	64	26.5	18
Europe ^a^	2 years	IGRA	5,020	58.2	5

^a^Not a high tuberculosis burden area. IGRA, interferon-gamma release assay; TST, tuberculin skin test.

## Mechanisms of early clearance of *Mycobacterium tuberculosis* infection

Initial recognition of aerosolized Mtb occurs in the lung, which leads to the production of pro-inflammatory cytokines, chemokines, and antimicrobial peptides (reviewed in 28, 29). What determines the outcome of exposure to Mtb? Is infection determined by the virulence of the microbe or more dependent upon host response? Is the immediate response in the respiratory tract important to host protection? Is infection determined by the ability of Mtb to establish a niche in a specific inflammatory microenvironment? Or does the quality, quantity, or organization of lung-resident lymphocytes influence early killing of Mtb and ultimate protection of the host?

### I. Early recognition of *Mycobacterium tuberculosis*

The airway is a complex immune organ and likely critical in determining the outcome of Mtb exposure. It exhibits anatomic and functional heterogeneity and contains a diverse array of mechanisms to prevent pulmonary infection, including mucociliary clearance, secreted antibodies, and antimicrobial proteins such as defensins ^28^. Although most research has focused on the alveolus as the initial site of infection, Mtb has ample opportunity to interact with the entire respiratory tract, like other aerosolized particulates. However, how these interactions result in clinically relevant outcomes such as infection and progression to disease is poorly understood. Reuschl *et al.* used polarized human lung epithelial cells in conjunction with myeloid cells to model these early interactions. Following Mtb infection, they mapped global transcriptomic changes in host cells. Interestingly, they found that myeloid cells could license epithelial cells, through interleukin-1 beta (IL-1β) and type I interferon, resulting in enhanced mycobacterial control ^30^. Additionally, following Mtb infection in the mouse, cathelicidins and related antimicrobial proteins produced by lung macrophages and epithelial cells were required for early clearance of infection ^31^. Mounting evidence suggests a role of humoral immunity, including antibodies, and antibody-responsive innate immune cells bearing Fc-receptors, in protective immunity to Mtb ^32– 35^. While studying patients already infected with Mtb, Lu *et al.* explored 70 different antibody features of total serum IgG from patients with LTBI compared with active TB. The authors’ data suggested an innate antibody Fc effector profile which led to the restriction of bacterial survival within macrophages ^33^. When evaluating HCs of patients with active TB, Chin *et al*. observed a different IgA antibody V-gene/D-segment repertoire in those without LTBI compared with those with LTBI, suggesting that a specific type of secretory IgA may promote mucosal protection from Mtb infection ^36^. A recently published genome-wide association study of persistently TST-negative HIV-positive HCs investigated mechanisms of resistance to infection ^22^. Here, HIV-positive contacts who resisted Mtb infection were postulated to have robust innate immunity. Sobota *et al.* found a significant association between TST negativity with a single-nucleotide polymorphism (SNP) at 5q31.1, which is located between *SLC25A48*, a mitochondrial amino acid transporter, and *IL-9*, a cytokine ^22^. IL-9 has been implicated in bronchial hyperreactivity and as a growth factor for mast cells and T cells ^22^. In an effort to search for biomarkers of stages of Mtb infection, Bark *et al.* evaluated HCs prospectively for differences in serum proteins over two years using mass spectrometry. They compared changes in host proteins in those who were persistently TST- and IGRA-negative, had LTBI, or had TB ^15^. Tissue integrity proteins, such as keratins, hornerin, lumican, and a component of the extracellular matrix, were more highly expressed in the serum of HCs who did not convert their TST and IGRA. It is interesting to speculate whether these proteins contribute to an essential early barrier against infection. Taken together, these data suggest a distinct role for the respiratory tract as the first line of defense against Mtb infection.

### II. Cell-intrinsic mechanisms of host resistance

Mtb virulence is a critical determinant of disease outcome following exposure. Although virulence must be broadly defined, one aspect is certainly the ability of the microbe to circumvent host immunity, such that the outcome reflects this complex interaction. It has been observed that the acquisition of adaptive immunity following infection with Mtb is delayed compared with other infections ^37, 38^. As a result, it has been postulated that Mtb has developed immune-evasive mechanisms to orchestrate this delay, allowing unrestricted replication in the lung. In fact, while using a low-dose aerosol model of TB in non-human primates, Gideon *et al*. asked whether there are differences in the immune response upon initial infection that determine the severity of the outcome ^39^. Through longitudinal blood transcriptome analysis, these data suggested that a highly orchestrated innate and adaptive immune response was crucial for containment of the bacilli.

To determine whether HCs who resist infection or have LTBI differ in their ability to produce innate cytokines in response to Mtb infection, Mahan *et al.* employed a whole blood enzyme-linked immunosorbent assay. Here, the responses to a panel of innate receptor ligands indicated no differences between HCs with or without LTBI ^25^. Although these data imply similar innate immune capabilities between those who remain TST-negative and those with LTBI, the authors state the need for more comprehensive study of innate protective mechanisms. Correspondingly, a subsequent study in HCs from the same district indicated that initial recognition of Mtb by distinct innate receptors contributes to controlling infection. Hall *et al.* performed comparative candidate immune gene SNP analysis in HCs with or without LTBI. They found SNPs in *NOD1*, *NOD2*, *SLC6A3*, *STAT1*, *IL12RB1*, *IL12RB2*, and *TLR4* that associated with a persistently negative TST ^24^. As these are intracellular and extracellular sensors of bacteria, this suggests the need for recognition of infection and activation of the host cell in resistance. Interestingly, NOD2 signaling is increased post-BCG vaccination for up to one year through a phenomenon called trained immunity ^40– 42^. Trained immunity is defined as resistance to reinfection and is thought to reflect persistent changes in innate pathways that can lead to memory. Further information from Manzanillo *et al.* supports intracellular sensing; phagosome acidification of Mtb can be triggered through another cytosolic surveillance pathway recognizing microbial cyclic dinucleotides and DNA ^43^. These data indicate that intracellular sensing of Mtb in the host cell is a crucial trigger to controlling the early infection.

A common feature of innate sensing of extracellular and intracellular Mtb is host cell activation, resulting in microbial killing. Host cells can directly kill Mtb by the production of antimicrobial proteins, reactive oxygen species, and acidification of the phagosome ^44, 45^. Acidification leads to autophagy, which in turn may help prevent or limit infection. Horne *et al*. found that a human SNP in *ULK1* was associated with infection. CRISPR/Cas9 gene editing to delete *ULK1* revealed that its deficiency in macrophages resulted in augmented Mtb growth, diminished tumor necrosis factor-alpha (TNF-α) production, and diminished autophagy ^17^. Other genes associated with the macrophage phagosome, such as *SLC11A1* or *natural resistance-associated macrophage protein 1* ( *NRAMP1*), have been linked to the development of TB previously ^46^. Although its full function remains to be elucidated, three studies have identified phagosome-associated *NRAMP1* expression as a protein associated with resistance to infection with Mtb. A seminal study of HCs in Uganda ^27^ used a genome-wide linkage analysis to reveal three regions associated with early clearance. Whereas the two most significant regions did not contain characterized genes, a third contained *NRAMP1*. Also, an *NRAMP1* polymorphism in the 3′ untranslated region (UTR) was more common in Venezuelan hospital workers who were persistently TST-negative than those with LTBI ^23^. Additionally, the transcriptome of host cell monocytes derived from persistently TST-negative HCs compared with those with LTBI has been explicitly interrogated by Seshadri *et al*. ^21^. Transcripts associated with histone deacetylase (HDAC) inhibition were enriched among persistently TST-negative HCs. Using chemical inhibition of HDACs in monocytes *in vitro*, the authors observed a role for HDACs in the early immune response to Mtb infection. As HDACs are associated with closed chromatin, this study would support a role for epigenetic programming of host cells in the control of Mtb infection.

Lastly, macrophages capable of non-inflammatory apoptosis and efferocytosis have inherently improved mycobacterial control by limiting intercellular spread ^47^. Recent work in animal models of natural transmission supports the hypothesis that augmented apoptosis can result in better control of Mtb infection. In this regard, cows that demonstrate resistance to *Mycobacterium bovis* infection have persistent TST negativity following herd exposure ^48– 50^. To explore these mechanisms in cattle, Wu *et al.* generated an *sp110*-TALEN-mediated knock-in cow strain. The gene *Ipr1/sp110* had been previously demonstrated to be associated with innate immunity to TB in mice ^51^. Sp110 is a nuclear body that permits apoptosis over inflammatory cell death in infected macrophages which decreases the viability of Mtb and limits intercellular spread ^52^. In a breakthrough finding, transgenic cattle where murine *sp110* is selectively expressed in macrophages were more resistant to *M. bovis* infection when a cow with active TB was placed in a herd ^53^. Macrophages from transgenic cattle were better able to “restrain” intracellular Mtb and preferentially died by apoptosis over necrosis *in vitro*. Although nitric oxide (NO) is traditionally thought to play a direct role in TB control, it is also shown to limit granulocytic inflammation and tissue damage in the context of Mtb infection, in part through modification of the inflammasome ^54^. Subsequently, it is interesting that Mtb-infected macrophages from TST-negative cattle were recently shown to produce more NO ^50^. Therefore, NO, a molecule made in abundance in human lung epithelial cells, has additional functions that may contribute to preventing infection with Mtb. These studies advocate that non-inflammatory cell death and innate antimicrobial responses derived from genetics or trained epigenetics of the host cell can contribute to controlling Mtb infection.

### III. Unconventional lymphocytes coordinate early lung inflammation to limit *Mycobacterium tuberculosis* infection

Longitudinal case control studies ( Table 1 and Table 2) would indicate that infection by Mtb can be controlled without an adaptive T-cell response ^18, 20, 25, 55– 58^. In addition to macrophages, lymphocytes, especially T cells, could orchestrate this response to Mtb infection. In addition to antigen-specific HLA-I and HLA-II restricted cells that can be reflected in a TST and IGRA, a variety of cells are capable of recognizing the Mtb-infected cell that may not be reflected in these assays. First, it is possible that a TST or IGRA test fails to reflect lung-resident memory T cells that do not circulate systemically ^59, 60^. Specifically, it has been argued that classically restricted tissue-resident memory T cells may recognize Mtb-infected cells and confer protection before the acquisition of a peripheral adaptive immune response ^61– 63^. Second, unconventional T cells, including CD1-restricted cells, MR1-restricted mucosal-associated invariant T (MAIT) cells, HLA-E/Qa-1-restricted cells, and γδ T cells, can also recognize Mtb infection and are poised at mucosal sites such as the lung ^59^. Each of these cell populations can also kill infected host cells through cytotoxic granules and produce cytokines in the context of Mtb. Mouse models of TB have been used to decipher whether these subsets have a protective role in the antimicrobial immune response. Using mice deficient in the antigen-presentation element, researchers have observed a role for MR1-restricted MAIT cells and Qa-1-restricted cells in the early response to Mtb infection ^64– 66^. In parallel experiments, group I CD1-restricted T cells specific to Mtb-derived lipids conferred protection in human CD1-transgenic mice ^67, 68^. Mouse models of TB do not suggest a non-redundant role for natural killer T cells or γδ T cells in a chronic TB setting, although they do show changes in frequency and phenotype in association with human Mtb infection ^69, 70^. Moreover, group-I CD1-, HLA-E-, and MR1-restricted T cells specific to Mtb antigen have been detected in individuals with active or LTBI infection ^68, 71– 75^. Collectively, these studies indicate a strong possibility that lung-resident T-cell populations play a role in the early immune response to Mtb in humans.

Lastly, natural transmission models of Mtb infection in guinea pigs have provided us with an important example of sterilizing adaptive immunity. Guinea pigs demonstrate a range of susceptibility to Mtb infection in natural transmission experiments ^76, 77^. A unique set-up of these experiments includes the direct exposure of guinea pigs to air from patients. They reflect many features of human immunity such as variable progression to disease. Additionally, some guinea pigs displayed the early clearance phenotype in that they became infected and then reverted to a negative TST. Sterilizing immunity was supported by the observation that the animals did not have granulomas or cultivable mycobacteria, and conversion was prevented by irradiating the air. Furthermore, administration of dexamethasone did not result in TB. As the prevalence of sterilizing immunity in humans is uncertain, future lessons from model systems may provide a paradigm of immune memory that may be crafted into future vaccine strategy or immune therapeutics.

## The immune race to control *Mycobacterium tuberculosis* infection

Viewed in aggregate, the studies presented here illustrate immune mechanisms that may facilitate early clearance or the eradication of Mtb before an adaptive immune response develops. In humans, our best indicators of early clearance come from longitudinal (two-plus years) analyses of HCs of TB patients or those residing in high TB burden areas, where exposure to Mtb and immune sensitization are carefully monitored. It is important to recognize that much of our knowledge of the host-defense mechanisms comes from the study of those with chronic disease. However, studies focusing on the early acute phases of Mtb infection have provided insights that support early and successful lung-resident immunity as key for preventing infection. As TB eradication will depend on preventing transmission, understanding immune correlates, such as improved macrophage function, mucosal antibodies, or enhanced recognition of the infected cell, will present new opportunities to prevent Mtb infection.

## Abbreviations

BCG, Bacille Calmette–Guérin; HC, household contact; HDAC, histone deacetylase; HLA, human leukocyte antigen; IGRA, interferon-gamma release assay; IL, interleukin; LTBI, latent tuberculosis infection; MAIT, mucosal-associated invariant T; Mtb, *Mycobacterium tuberculosis*; NO, nitric oxide; NRAMP1, natural resistance-associated macrophage protein 1; SNP, single-nucleotide polymorphism; TB, tuberculosis; TST, tuberculin skin test; WHO, World Health Organization
